# Invasion and rapid adaptation of guppies (*Poecilia reticulata*) across the Hawaiian Archipelago

**DOI:** 10.1111/eva.13236

**Published:** 2021-05-04

**Authors:** William C. Rosenthal, Peter B. McIntyre, Peter J. Lisi, Robert B. Prather, Kristine N. Moody, Michael J. Blum, James Derek Hogan, Sean D. Schoville

**Affiliations:** ^1^ Center for Limnology University of Wisconsin‐Madison Madison WI USA; ^2^ Department of Botany University of Wyoming Laramie WY USA; ^3^ Department of Natural Resources Cornell University Ithaca NY USA; ^4^ Department of Evolution, Ecology, and Organismal Biology University of California Riverside Riverside CA USA; ^5^ Department of Ecology and Evolutionary Biology University of Tennessee Knoxville Knoxville TN USA; ^6^ The ByWater Institute Tulane University New Orleans LA USA; ^7^ Oak Ridge National Laboratory Oak Ridge TN USA; ^8^ Department of Life Sciences Texas A&M University‐Corpus Christi Corpus Christi TX USA; ^9^ Department of Entomology University of Wisconsin‐Madison Madison WI USA

**Keywords:** biological adaptation, Hawai‘i, introduced species, molecular evolution, population genomics

## Abstract

How much does natural selection, as opposed to genetic drift, admixture, and gene flow, contribute to the evolution of invasive species following introduction to a new environment? Here we assess how evolution can shape biological invasions by examining population genomic variation in non‐native guppies (*Poecilia reticulata*) introduced to the Hawaiian Islands approximately a century ago. By examining 18 invasive populations from four Hawaiian islands and four populations from the native range in northern South America, we reconstructed the history of introductions and evaluated population structure as well as the extent of ongoing gene flow across watersheds and among islands. Patterns of differentiation indicate that guppies have developed significant population structure, with little natural or human‐mediated gene flow having occurred among populations following introduction. Demographic modeling and admixture graph analyses together suggest that guppies were initially introduced to O‘ahu and Maui and then translocated to Hawai‘i and Kaua‘i. We detected evidence for only one introduction event from the native range, implying that any adaptive evolution in introduced populations likely utilized the genetic variation present in the founding population. Environmental association tests accounting for population structure identified loci exhibiting signatures of adaptive variation related to predators and landscape characteristics but not nutrient regimes. When paired with high estimates of effective population sizes and detectable population structure, the presence of environment‐associated loci supports the role of natural selection in shaping contemporary evolution of Hawaiian guppy populations. Our findings indicate that local adaptation may engender invasion success, particularly in species with life histories that facilitate rapid evolution. Finally, evidence of low gene flow between populations suggests that removal could be an effective approach to control invasive guppies across the Hawaiian archipelago.

## 1 INTRODUCTION

The balance between counteracting evolutionary forces can determine whether a non‐native species flourishes or fails following introduction to a new environment. While there are many examples of rapid evolution in invasive species (Prentis et al., 2008), it remains unclear how genetic drift, admixture, and natural selection integrate to shape invasions (Colautti & Lau, 2015). Theory lays out some expectations; for instance, adaptation to novel environments could permit establishment and spread of non‐native species (Lavergne & Molofsky, 2007; Lee et al., 2007) despite initial constraints like reduced genetic diversity (Baker & Moeed, 1987) and small population sizes (Allendorf & Lundquist, 2003). In addition, standing genetic variation can be elevated through repeated rounds of introduction, admixture from multiple source populations, or even interspecific hybridization (Ellstrand & Schierenbeck, 2000; Kolbe et al., 2004; Roman & Darling, 2007). The potential for adaptive evolution in invasive populations may also be elevated by the species composition of the invaded community (Faillace & Morin, 2016), and the ability to draw on plasticity or epigenetic sources of variation (Bock et al., 2015). Given this range of potential influences on populations after establishment, reconstructing the history of introductions and subsequent evolutionary dynamics can offer valuable insight on the factors that differentiate successful and failed invasions.

Understanding the role of evolutionary processes in invasions can also guide efforts to constrain and eliminate non‐native species. For instance, reconstructing the history and sequence of an invasion can inform managers of natural conditions or human activities that facilitate further spread. Frameworks for identifying species with extraordinary invasive potential could be improved by including information on a species' capacity for adaptation as there is good evidence for species undergoing rapid evolution following introduction to a novel environment, often involving traits associated with colonization and demographic expansion (Whitney & Gabler, 2008). Accounting for ecological drivers of rapid evolution could also allow risk assessments to focus on sites where postintroduction adaptive potential is greatest. Given the predicted increase of biological invasions due to globalization (Early et al., 2016), advancing management practices by accounting for the evolutionary potential of invasive species might prove critical for conserving biodiversity and ecosystem services.

Examining species with a demonstrated capacity for rapid evolution, such as guppies (*Poecilia reticulata* Peters 1859), can help elucidate how different forces and associated factors (e.g., number of introduction events and founding population size) influence invasion success. Guppies not only exhibit a number of traits that contribute to establishment and population growth following introduction (e.g., a short generation time, a viviparous life history, and sperm storage; Deacon et al., 2011), but also serve as a model system for the study of rapid adaptation. Experiments in their native range of South America offer evidence of rapid life‐history evolution in response to shifts in ecological conditions like predation regime (Gordon et al., 2009; Reznick et al., 1990, 1997). Other factors are known to influence guppy evolution such as ecosystem productivity (Reznick et al., 2001) and prey availability (Zandonà et al., 2011). As a complement to decades of work on guppy evolution in nature, the availability of an annotated reference genome for guppies (Künstner et al., 2016) also can be leveraged to investigate evolutionary change in non‐native populations.

Non‐native populations of guppies now occur worldwide (Chandra et al., 2008; Deacon et al., 2011; Liang et al., 2006), including in the Hawaiian Islands where conditions may have fostered adaptive evolution. Historical records suggest that guppies were initially introduced from an unknown source to O‘ahu approximately 100 years ago for mosquito control (Brock, 1960). Guppies have since become widespread and now occur in the majority of watersheds across the archipelago (Moody, Gagne, et al., 2017). Following initial introduction, movement of individual guppies within and between watersheds is challenging without human aid due to barriers such as waterfalls and the marine environment. Guppies are subject to a range of biotic and abiotic conditions in stream ecosystems across the archipelago, reflecting differences in landscape characteristics, predation pressures, and nutrient availability (Lisi et al., 2018; Moody, Gagne, et al., 2017). The potential overlay of multiple selective pressures raises the intriguing possibility of differentiation across a geographic mosaic, with natural selection acting on large but isolated guppy populations.

In this study, we explored the nature of evolutionary forces acting on non‐native populations of guppies in the Hawaiian Islands. We did so by evaluating population genomic variation within and among 18 populations on four islands, with reference to four populations in the native range. This allowed us to identify population structure between watersheds and among islands to infer the amount of gene flow between populations. We also reconstructed the invasion history and historical demography of guppies across the archipelago, with the aim of deducing the number of source populations introduced to the archipelago. Lastly, we tested for signatures of local adaptive evolution in response to predators, nutrient regimes, and landscape characteristics.

## 2 MATERIALS AND METHODS

### 2.1 Sampling, DNA extraction, and genotyping by sequencing

**FIGURE 1:**
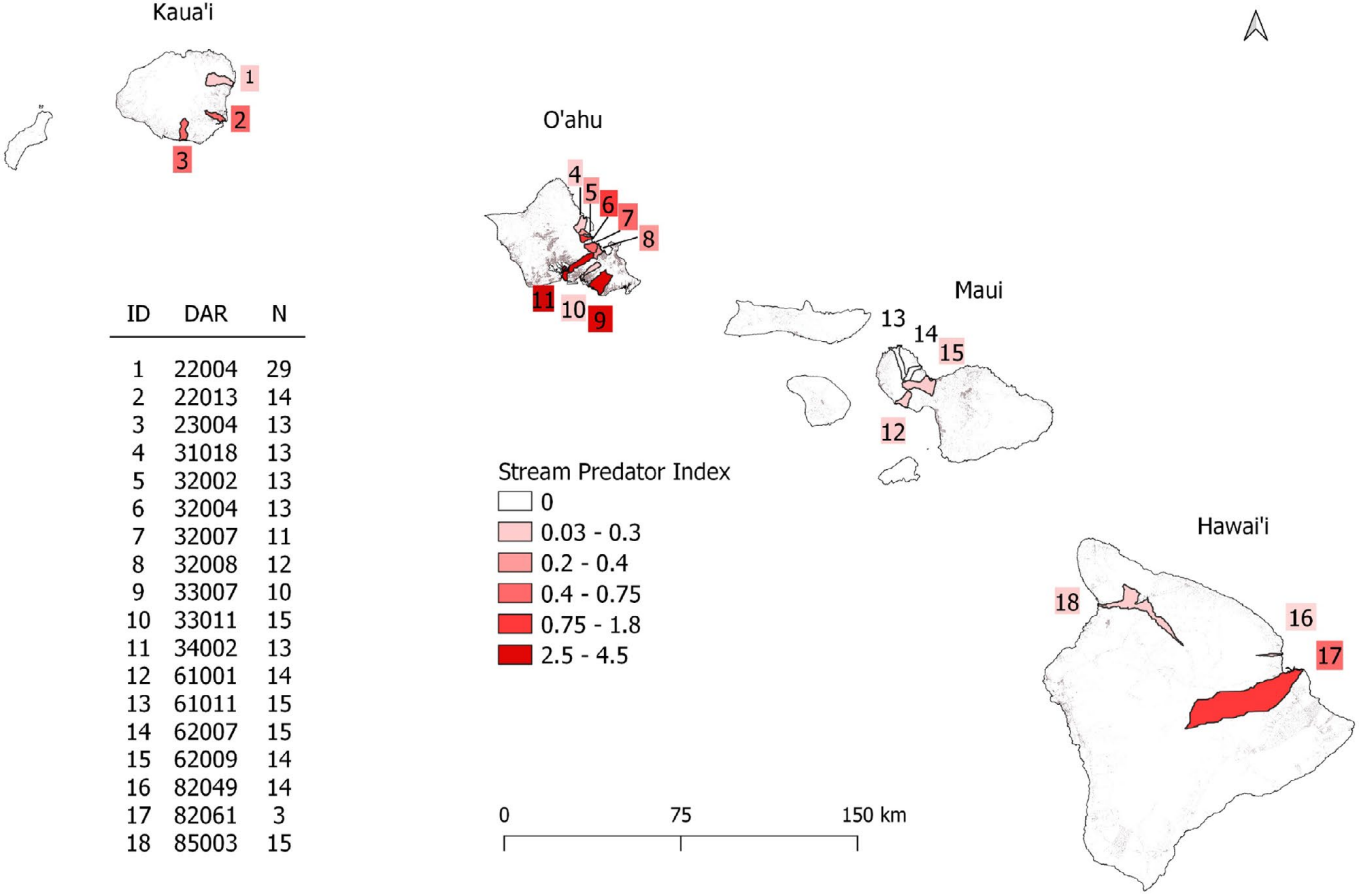
Map of the Hawaiian archipelago with sampled watersheds colored by the predator density metric used in the LFMM analyses. The predator density value for each watershed was estimated via snorkel surveys done in the upper, middle, and lower reaches of the watershed. The observed predator densities were then weighted by predator size to integrate differences in predation risk arising from both predator identity and density. Watersheds are labeled with a number that corresponds to the adjacent table noting corresponding watershed names and number of individuals (*N*) sampled from each watershed

Fish from 18 watersheds across four islands of the Hawaiian archipelago were hand‐netted in 2009, with supplemental collections made in March 2016 (Figure 1, Table S1). Trinidadian fish were hand‐netted in June 2017 from the Quare, Aripo, and Yarra rivers (and river basins, respectively), which harbor all major evolutionary lineages on the island (Willing et al., 2010). Notably, the Quare and Aripo samples were collected from “high predation” guppy populations, whereas the Yarra samples were collected from a “low predation” population. High‐predation populations co‐occur with the large predator *Crenicichla alta*, whereas low predation populations only co‐occur with the smaller omnivore *Rivulus hartii* (Reznick et al., 1997). Thus, our Trinidadian collections efficiently capture a representative sample of genetic variation present on the island. The Venezuelan samples were hand‐netted from Rio Las Marias (Orinoco Basin) in 2001. All samples were stored in 95% ethanol. Sample sizes ranged from 3 to 29 individuals across all locations (Figure 1, Table S1).

DNA was extracted from caudal fin and caudal peduncle tissue using the Qiagen DNeasy Blood and Tissue Kit. The quantity of extracted DNA was assayed using a Qubit Flourometer with dsDNA high‐sensitivity kits. The integrity of the DNA was verified through gel electrophoresis visualization. Library preparation was done at the University of Wisconsin‐Madison Biotechnology Center using genotyping by sequencing methods (Elshire et al., 2011) with ApeKI as a restriction enzyme. Sequencing was also done at the University of Wisconsin‐Madison Biotechnology Center on an Illumina HiSeq 2500.

### 2.2 SNP genotyping and quality control

The resultant 100 base pair single‐end reads were demultiplexed, filtered for read quality, and filtered for adapter contamination using the process_radtags function of stacks v1.44 (Catchen et al., 2013). These reads were then aligned to the guppy reference genome (NCBI accession GCF_000633615.1, Künstner et al., 2016) using bowtie2 (Langmead & Salzberg, 2013). Single nucleotide polymorphisms (SNPs) were called using the reference aligned workflow (pstacks, cstacks, sstacks, populations) outlined in the user manual. SNPs were removed if the minor allele frequency was 0.03 or less. Six individuals with >75% missing data were excluded from the dataset. As some samples exhibited highly divergent multilocus genotypes, the mitochondrial control region was sequenced to assess species assignment for a subset of samples. Approximately 490 base pairs of the control region were amplified and sequenced using primers L15926 and H16498, with accompanying protocols developed by Ptacek and Breden (1998), for eight samples (representing *P*. *reticulata* from the Trinidadian Yarra population and seven distinct population clusters from the Hawaiian archipelago selected using genome‐wide SNPs, see below; NCBI accessions MN922313–MN922320). Subsequent blastn searches of the control region (Altschul et al., 1990) revealed that two groups of samples, representing two genome‐wide SNP clusters that contained 44 individuals, were most likely a different species (*Gambusia affinis* and *Poecilia orri*). These samples were excluded from subsequent analyses. Loci with greater than 50% missing data were removed, and the remaining missing data were imputed using BEAGLE v4.1 (Browning & Browning, 2016). Nucleotide diversity, *π*, and expected heterozygosity, *H*_e_, were calculated for each population using stacks v1.44.

### 2.3 Analysis of population structure

We used a combination of approaches to assess the nature of genomic variation within and among the study sites. Population genomic variation was first visualized through principle components analysis (PCA) using the pca function of the LEA package (Frichot & François, 2015) in R (R Core Team, 2013). Scree plots were used to determine when the explanatory power of additional eigenvectors declined sharply. PCA also was performed on the pre‐imputation dataset to confirm that general patterns of population structure were minimally affected by the imputation process. The effect of imputating missing genotype data was quantified by calculating correlations between PC1 and PC2 scores with pre‐imputation missing genotype data proportions for each individual. We also performed a PCA of only Hawaiian individuals. A clustering algorithm was then used to examine population structure assuming the study populations experienced no genetic drift from their ancestral populations. The cluster analysis was implemented using the sNMF function of LEA. The sNMF algorithm was initialized with cluster numbers ranging from 1 to 12, each with 10 replicates, and the best model was determined by minimizing the cross‐entropy among clusters. Pairwise *F*_ST_ values were estimated between each population with the R package BEDASSLE (Bradburd et al., 2013), which uses Weir and Hill's *θ* to assess divergence between populations (Weir et al., 2002). Finally, an analysis of molecular variance (AMOVA) was performed on the Hawaiian guppy populations using the R package poppr (Kamvar et al., 2014) to determine which levels (e.g., individual, population, island) of segregation contribute most to overall genomic variance.

### 2.4 Analysis of invasion history

TreeMix (Pickrell & Pritchard, 2012) was used to generate a maximum likelihood admixture graph of all populations with the Trinidadian Yarra river population selected as the outgroup. This approach estimates relative drift among a set of populations, with split patterns and branch lengths reflecting population divergence history. It assumes that population divergence generally reflects a tree‐shaped topology, which can lead to significant biases in results when there is substantial admixture. We did not utilize the migration event estimation feature of TreeMix, as it can be challenging to use the maximum likelihood optimization in TreeMix to infer the correct topology in combination with gene flow events when many populations are included in the analysis. Instead, the potential for admixture across islands was tested with *D*‐statistics (Green et al., 2010) calculated using the Dtrios program in Dsuite (Malinsky et al., 2021). *D*‐statistics were evaluated for all possible trios of all sampled Hawaiian watersheds, with all native range individuals used as an outgroup. Jackknife block sizes of 100, 200, 1000, and 2000 SNPs were used. The resulting *p*‐values were corrected for multiple testing bias via the p.adjust function in R.

Stairway plots for each population were created to examine the chronology of introduction and relative population size changes for each Hawaiian watershed. The software dadi (Gutenkunst et al., 2009) was used to create folded SNP frequency spectra for each watershed, which were then used to generate the input files for stairway plot v2.1 (Liu & Fu, 2015). This function uses a multi‐epoch model to calculate the expected composite likelihood of the SNP frequency spectrum, which can be used to estimate recent changes in population size. Analyses were based on a generation time estimate of 3.5 generations per year (Magurran, 2005) and a mutation rate estimate of 4.89 × 10^−8^ mutations per nucleotide per generation (Künstner et al., 2016). Sequence length values (including variant and nonvariant sites) for each population were obtained from the sumstats_summary file from the stacks populations output. The Venezuelan Rio Las Marias population and one population from the island of Hawai‘i were excluded from these analyses due to small sample sizes (*n* < 8). The median, upper, and lower 95% confidence interval bound estimates of effective population size (*N*_e_) from >30 years ago were calculated to provide a point estimate of *N*_e_ (with 95% confidence intervals) for each population that would account for changes in *N*_e_ through time.

**FIGURE 2:**
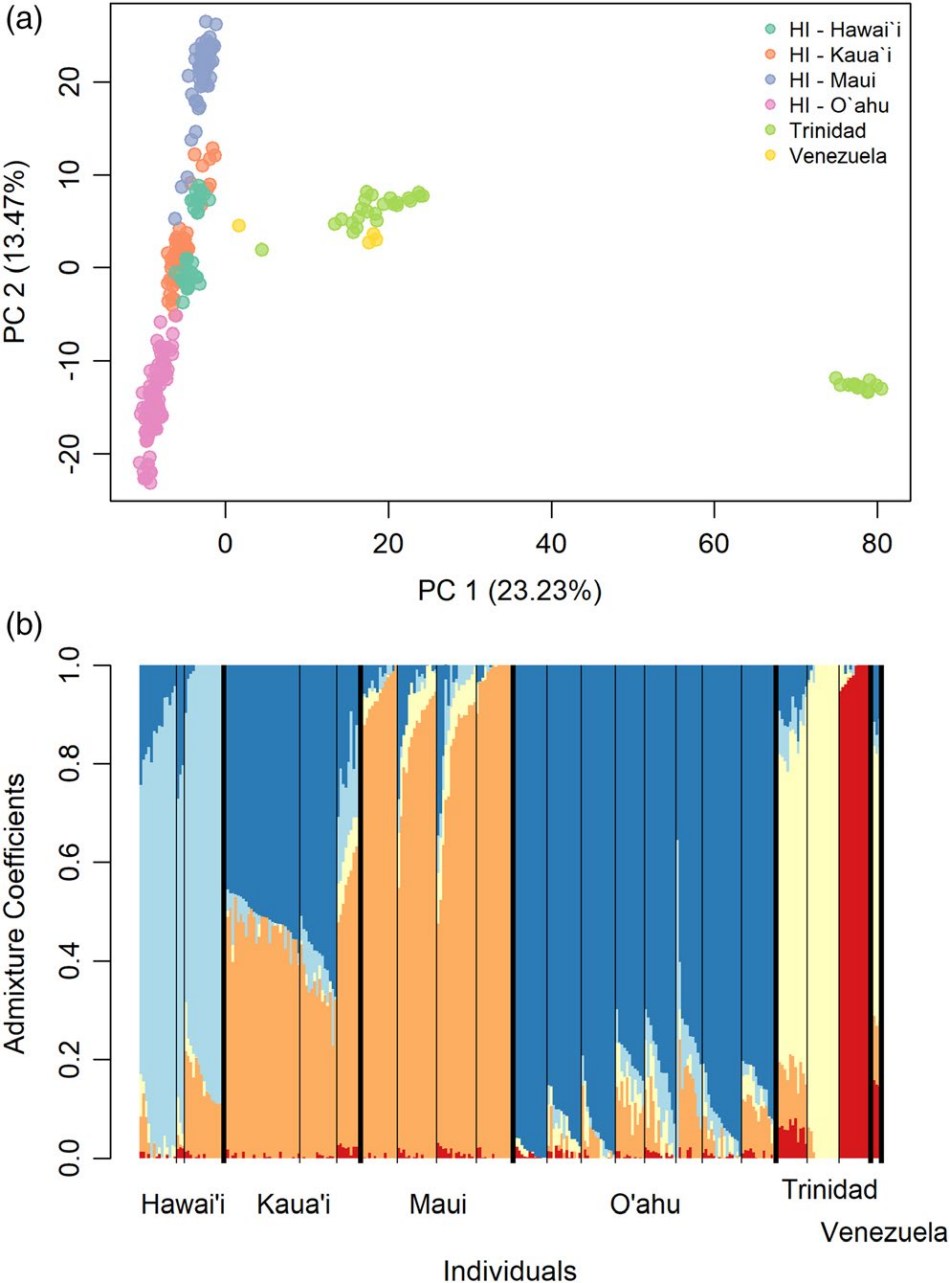
(a) Principle components analysis (PCA) results with individuals colored by island or region of origin. PC1 and PC2 explain 23% and 13% of the total variance, respectively. Intermediate individuals in the plot had higher‐than‐average amounts of missing data before genotype imputation. (b) Results from sNMF admixture analysis with *K* = 5 clusters. Each thin black line separates watersheds within an island or region, and each island or region is separated by a thick black line

Demographic models were constructed using dadi (Gutenkunst et al., 2009) to infer the number of source populations introduced to the archipelago. The PCA (Figure 2a), sNMF (Figure 2b, Appendix S2, Figure S1), and *D*‐statistic results indicated that populations on Maui and O‘ahu were the most divergent by island and that samples from Kaua‘i and the island of Hawai‘i exhibit admixed ancestry drawing from both Maui and O‘ahu. Because of this, we reasoned that the introduction of multiple source populations to the archipelago could be determined by estimating the divergence time between Maui and O‘ahu populations. If only one source was introduced to the archipelago, the divergence time between Maui and O‘ahu would be estimated near 100 years ago (i.e., the approximate date when guppies were recorded as having been introduced to the archipelago). If variation from another source population was contributing to the divergence between Maui and O‘ahu, we would instead expect to find evidence of a deeper ancestral divergence time among natural source populations. It is important to note that our approach is not designed to detect mixture among very recently diverged native source populations, but this scenario would be unlikely to contribute substantial novel genomic diversity to an invading population (i.e., recently diverged populations share common alleles found in their ancestor and would have few private alleles). We therefore constructed two nested models; both included Maui and O‘ahu splitting from an ancestral population, and with one including continuous migration between populations after divergence (Figure S2). Migration was allowed to be asymmetrical. A model with migration was included because migration between the populations, if supported over the no migration model, would likely impact the estimated divergence time. Each island was represented by the population with the largest sample size (Maui 62007, O‘ahu 33011, *n* = 15). The residuals of each model were examined for normality before model fits were compared using an adjusted log‐likelihood ratio test (from the LRT_adjust function in dadi), which accounts for linkage between loci. Parameter estimates were obtained using the log_lbfgsb algorithm in dadi, and the stability of parameter estimates was confirmed via multiple rounds of model fitting. The 95% confidence intervals for each parameter in the best‐fit model were obtained by generating 100 bootstrap replicate datasets and fitting the model to each one. Bootstrapping was done over the SNPs found in each chromosome or scaffold. The 95% confidence interval for each parameter was defined as *α* ± 1.96 * *β*, where *α* is the parameter's value from the original model and *β* is the standard deviation of that parameter's bootstrap distribution. This approach allowed for uncertainty to be calculated for each parameter estimate while accounting for linkage between loci. Divergence time estimates were converted from dadi's time units (2*N*_ref_ generations, where *N*_ref_ = *θ*/(4 × *μ* × *L*)) using a mutation rate (*μ*) of 4.89 × 10^−8^. Sequence length (*L*) was again taken from the sumstats_summary file from the stacks populations output, but was modified to reflect the effects of SNP filtering and linkage on effective sequence length. The original length output was multiplied by the number of SNPs in the filtered dataset (not within 100 bp downstream of another SNP) divided by the number of SNPs in the unfiltered VCF (resultant value of 0.0216). The parameter *θ* was estimated from the best‐fit model using the optimal_sfs_scaling function in dadi. Time was converted to years using a generation time of 3.5 generations per year (Magurran, 2005).

### 2.5 Environmental data and environmental association analysis

The influence of environmental factors on genomic variation was tested by first identifying key abiotic and biotic conditions that define Hawaiian streams. Five environmental variables were calculated from the results of Lisi et al. (2018) to capture representative differences in natural environmental pressures, anthropogenic environmental pressures, and potential predation pressures acting on guppy populations. As proxies for human transformation of land cover and natural variation in watershed character across the Hawaiian archipelago, we used the first and second principal components from a PCA of six major classes of land use and four landscape variables characterized for each watershed (Lisi et al., 2018). Landscape PC1 primarily reflects variable loadings describing forest and urban cover, whereas landscape PC2 primarily reflects watershed area, slope, and percent agriculture. To summarize stream water biogeochemistry, we used the first and second principal components from a PCA of eight water quality metrics (Lisi et al., 2018). Water quality PC1 primarily reflects correlates of human‐derived nitrogen loading, whereas water quality PC2 represents natural variation in dissolved phosphorus. To address biotic differences, our fifth environmental predictor was an index of potential predation pressure based on the weighted density of piscivorous fishes in each stream. Densities of predatory species with a small body size, such that they would not be able to eat adult guppies easily (*Awaous stamineus*, *Poecilia* sp., and *Xiphophorus helleri*), were assigned a weighting of half the per capita impact of larger predatory species (*Amatitlania nigrofasciata*, *Eleotris sandwicensis*, *Clarias fuscus*, *Hemichromis elongatus*, *Micropterus dolomieu*, and *Oreochromis* spp.). This weighting approach to aggregating across diverse species of potential predators was intended to integrate differences in predation risk arising from both predator identity and density. A PCA of the environmental data for each population included in this study can be found in Figure S3.

To determine the relative influence of the selected environmental variables on invasive guppies, we examined associations with overall genetic diversity and per locus SNP genotype frequency. Genetic diversity for each population was measured as expected multilocus heterozygosity (*H*_e_), and Pearson correlations were used to test for association of *H*_e_ with each environmental variable (Blum et al., 2012). To draw more specific inferences about responses to selection, environmental association tests (Rellstab et al., 2015) were conducted with genotype frequencies of each SNP. First, Hawaiian populations were scanned for environment‐associated SNPs using a latent factor mixed‐model from the R package LFMM (Frichot et al., 2013), which corrects for confounding effects of population structure. The calibrated *p*‐values from LFMM were visualized to confirm a relatively uniform *p*‐value distribution before multiple testing bias was corrected using the qvalue function in the qvalue R package (Storey, 2002). SNPs were considered significantly correlated if the corrected *p*‐value was <0.05.

Correlated loci were further evaluated in a series of validation steps. First, populations on the high and low ends of each environmental variable range were compared for allele frequency shifts across all loci using Fisher's exact tests. The number of environment‐associated and nonassociated loci with extreme (at or above the 0.95 quantile of all shifts) and nonextreme frequency shifts were used to determine whether environment‐associated loci were more likely to exhibit an extreme allele frequency shift across populations with disparate environmental conditions. Second, we compared the allele frequencies of loci associated with our predation pressure metric between Hawaiian populations with the highest predation metric score and the Trinidadian high‐predation Aripo and Quare populations. Finding similar allele frequencies would suggest that Hawaiian and Trinidadian guppies exhibit convergent responses to greater predation pressure. Lastly, candidate loci from the environmental association tests were mapped on to the annotated genome of *P*. *reticulata*. Only those SNPs within a gene coding region or within 200 bp upstream or downstream of a coding region were retained as putative functional variants. Ontology information for genes associated with environmentally correlated SNPs was obtained from the UniProt database (The UniProt Consortium, 2019).

## 3 RESULTS

### 3.1 Analysis of population structure

After filtering for minor allele frequency and missing data, genotyping by sequencing produced a dataset comprising 12,254 SNPs across 282 individuals. *H*_e_ values ranged from 0.0007 in Venezuela's Rio Las Marias to 0.0055 in O‘ahu population 32004 (Table S1). Nucleotide diversity (*π*) ranged from 0.0011 in the Trinidadian Yarra River population to 0.0059 in O‘ahu population 32004 (Table S1).

Both the sNMF‐based cross‐entropy and the PCA‐based scree plot supported *K* = 5 as the optimal number of clusters when considering all samples. PC1 and PC2 explained 23% and 13% of genomic variance, respectively. Two individuals that were recovered in an intermediate region of the PCA plot (Figure 2a) had higher‐than‐average amounts of missing data, but not enough to be removed during the filtering process. While the proportion of missing data before genotype imputation was not correlated with PC2 score, it was weakly but significantly correlated with PC1 score (Pearson's *r* = 0.21, *p* = 0.0003). This is likely due to the higher average proportion of missing data for the native range samples relative to the Hawaiian samples. PCA results with nonimputed genotypes closely resembled those from the analysis with imputed genotypes (Figure S4), and PCA results from the analysis of only Hawaiian individuals show a similar pattern of Maui and O‘ahu populations being most divergent from one another (Figure S5). PCA and sNMF results indicate that there is stronger differentiation among native range populations than among Hawaiian populations. Results for sNMF with values from *K* = 2 to *K* = 5 corroborate this finding (Figure S1). The Aripo River population clustered more closely with Venezuelan conspecifics than with other watersheds on Trinidad. Broadly, populations from the same island of the Hawaiian archipelago clustered together, with the exception of Kaua‘i population 22004. AMOVA partitioned 69% of the variance to within individuals, while variation between populations within islands corresponded to 15.2% of the overall variation. Variation between islands and between individuals within a population constituted 9% and 6.8% of the total variance, respectively. Pairwise *F*_ST_ values ranged from 0.07 to 0.39 between Hawaiian populations, further illustrating the presence of population genomic structure (Table S2).

### 3.2 Analysis of invasion history

**FIGURE 3:**
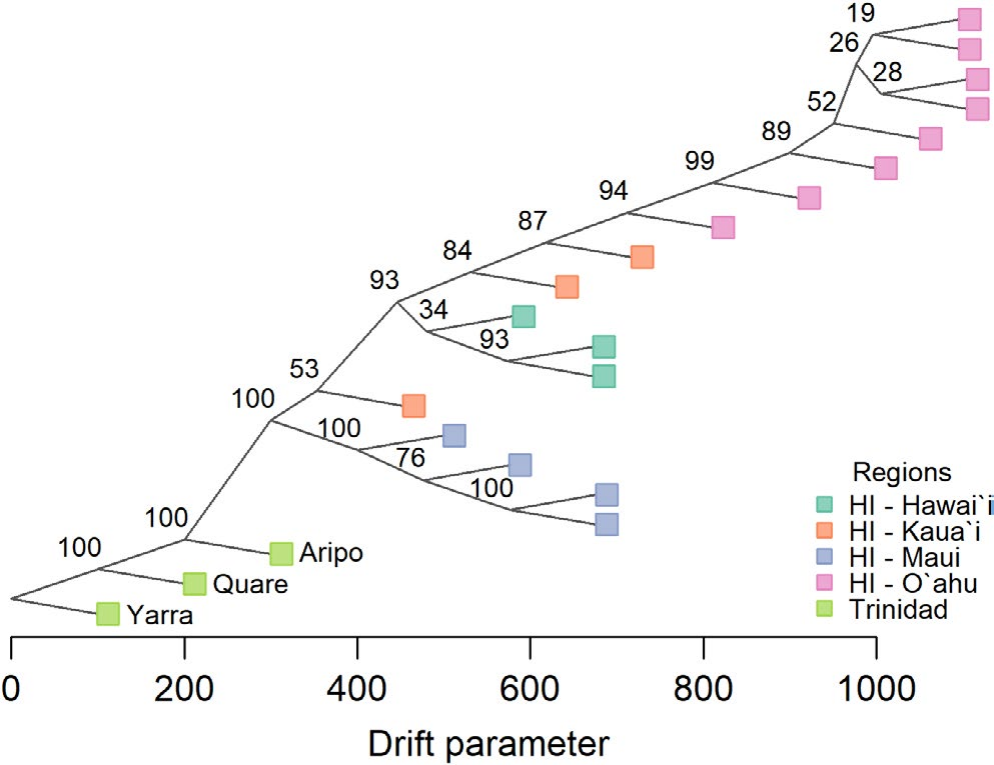
TreeMix admixture graph results. Line length denotes the amount of shared genetic drift between populations, and numbers beside nodes indicate the bootstrap support out of 100 for that node placement. The Yarra population was set as an outgroup

Populations on each Hawaiian island were estimated to be more closely related to each other than to populations from other islands or the native range in TreeMix, with the exception of one population from Kaua‘i (22004; Figure 3). *D*‐statistics revealed evidence of excess allele sharing and a lack of tree‐ness (i.e., simple bifurcating divergence without admixture) for 49 of 816 tested watershed trios. For trios comprising watersheds from three different islands, 21 of 300 unique trios had significantly nonzero *D*‐statistic values (Table 1). Trios with significantly nonzero *D*‐statistics included every sampled watershed except for watersheds 23004 (Kaua‘i), 32007 (O‘ahu), 31018 (O‘ahu), and 61011 (Maui). Of the 21 unique trios with significant *D* values, 11 included watersheds from the islands of Kaua‘i, O‘ahu, and Maui, seven included watersheds from the islands of Kaua‘i, O‘ahu, and Hawai‘i, and three included watersheds from the islands of O‘ahu, Maui, and Hawai‘i. No significant trio included watersheds from Kaua‘i, Maui, and Hawai‘i. *D*‐statistic results were similar across all tested jackknife block sizes.

**TABLE 1:** *D*‐statistics values calculated following Malinsky et al. (2021), with adjusted *p*‐values based on a jackknife block size of 1000 SNPs. *D*‐statistics were calculated for all possible trios of all possible watersheds, but only those including watersheds from three different islands and significantly nonzero *D*‐statistics are shown here

Watershed 1	Watershed 2	Watershed 3	*D*‐statistic	*p*‐value
Hawai‘i 82061	O‘ahu 33011	Kaua‘i 22004	0.074362	0.001172
Kaua‘i 22004	O‘ahu 33007	Hawai‘i 82049	0.068729	<0.0001
Kaua‘i 22004	O‘ahu 33007	Hawai‘i 82061	0.059628	0.000243
Kaua‘i 22004	O‘ahu 33011	Hawai‘i 82049	0.066055	<0.0001
Kaua‘i 22013	O‘ahu 33007	Hawai‘i 82049	0.055196	0.009737
Kaua‘i 22013	O‘ahu 33007	Hawai‘i 82061	0.049529	0.031205
Kaua‘i 22013	O‘ahu 33011	Hawai‘i 82061	0.071219	0.000197
Maui 62007	Hawai‘i 85003	O‘ahu 32004	0.069335	0.023167
O‘ahu 32002	Kaua‘i 22004	Maui 61001	0.084096	0.002592
O‘ahu 32002	Kaua‘i 22004	Maui 62009	0.079165	<0.0001
O‘ahu 32002	Kaua‘i 22013	Maui 61001	0.088664	0.008096
O‘ahu 32002	Kaua‘i 22013	Maui 62009	0.08292	0.001547
O‘ahu 32008	Hawai‘i 82049	Maui 62009	0.043996	0.042604
O‘ahu 32008	Kaua‘i 22004	Maui 62007	0.059129	0.013418
O‘ahu 32008	Kaua‘i 22004	Maui 62009	0.081653	0.024207
O‘ahu 32008	Kaua‘i 22013	Maui 61001	0.092065	<0.0001
O‘ahu 32008	Kaua‘i 22013	Maui 62007	0.053167	0.003693
O‘ahu 32008	Kaua‘i 22013	Maui 62009	0.084764	0.00013
O‘ahu 33007	Hawai‘i 85003	Maui 62009	0.044834	0.017081
O‘ahu 33011	Kaua‘i 22013	Maui 61001	0.07099	0.000253
O‘ahu 34002	Kaua‘i 22013	Maui 62009	0.056355	0.013071

**FIGURE 4:**
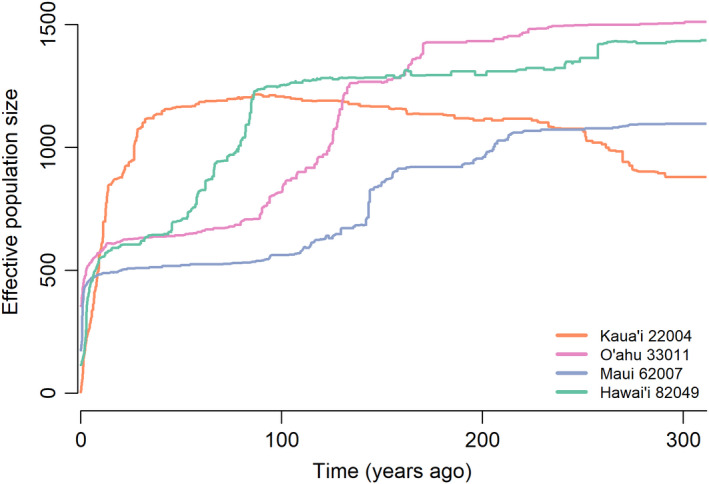
Change in effective population sizes over time inferred from stairway plots for the populations with the largest number of samples from each Hawaiian island. Lines indicate the median estimate of *N*_e_; see Figure S6 for change in *N*_e_ over time in each population, including confidence intervals. The O‘ahu and Maui populations shown here are the same as those used in the demographic modeling analysis

The stairway plot analysis detected a signature of a population bottleneck around 100 years ago in all but five of the Hawaiian guppy populations (Kaua‘i populations 22004 and 23004, O‘ahu populations 32002, 32007, and 33007) (Figure 4), but not in the native range populations (Figure S6). The timing of the inferred bottleneck corresponds reasonably well with the supposed date of guppies being introduced to the archipelago (Brock, 1960). We also detected a dramatic decrease in population size in the Trinidadian Yarra River population prior to 500 years ago. Median *N*_e_ estimates ranged from 244 individuals in an O‘ahu population (31018) to 1897 individuals in a Kaua‘i population (23004). The average median *N*_e_ estimate for all Hawaiian populations was 1087 individuals, with average 95% confidence interval bounds of 606 (lower) and 1913 (upper) individuals (Figure S6).

Demographic models provided support for introductions to the Hawaiian archipelago deriving from one source population. The log‐likelihood ratio test indicated that the addition of gene flow parameters did not improve the model fit; the models with and without gene flow had log‐likelihoods of −1806.563 and −1806.556, respectively (adjusted likelihood ratio test *p*‐value of 1.0). Residuals between the best‐fit model and data can be found in the supplementary materials (Figure S7). The model without gene flow estimated the divergence time between the O‘ahu and Maui populations at 0.010 units of $2N_{e_{A}}$ generations (2.5% confidence interval bound at 0.00758, 97.5% confidence interval bound at 0.1329). Converting divergence times to real‐time provides an estimated divergence time for Maui and O‘ahu of 86.63 years (2.5% confidence interval bound at 62.93 years, 97.5% confidence interval bound at 110.36 years).

### 3.3 Environmental association analysis

Genetic diversity, as measured by *H*_e_, was only correlated with potential predation pressure (*r* = 0.5955, *p*‐value = 0.0092). We detected statistical associations between genotype frequencies and at least one environmental predictor for 121 SNPs (Table 2). Of these 121 loci, 113 were correlated with just one environmental variable—most often landscape PC2 (watershed slope, area, and percent agriculture). Only one SNP locus was associated with water quality PC1 (nitrogen), and 13–19 loci were associated with each of the other environmental predictors. According to Fisher's exact tests, loci associated with landscape PC1 and water quality PC1 did not exhibit an enriched number of extreme allele frequency shifts, whereas loci associated with other environmental variables did (Table 2). Notably, 10 of 19 loci that were associated with the predation pressure index in the Hawaiian populations had allele frequencies of 0.00 in both Trinidadian high‐predation populations (Aripo and Quare Rivers). In contrast, the Hawaiian populations exhibited much wider variation; allele frequencies at these 19 loci ranged from 0.13 to 0.855, where the two O‘ahu populations with the highest predation pressure scores also had the highest allele frequencies in the archipelago.

**TABLE 2:** Environment‐associated loci identified through LFMM genomic analysis, with environmental variables from Lisi et al. (2018)

Environmental variable	Components of environmental variable	Number of associated loci	Fisher's exact test *p*‐value
Landscape PC1	Forested and urban land cover	13 (1)	0.487
Landscape PC2	Watershed slope, size, and percent agriculture	72 (19)	<0.0001
Nutrient PC1	Anthropogenic nitrogen input	1 (1)	0.0806
Nutrient PC2	Dissolved phosphorus	15 (6)	<0.0001
Predator density	Size‐weighted predator density	19 (6)	0.0002

Note

Values in parentheses indicate the number of loci associated with that environmental variable that also exhibited an extreme allele frequency shift, defined as an allele frequency shift in the 0.95 quantile of all allele frequency shifts between the populations with the highest and lowest scores for the respective environmental variable. The vast majority of correlated loci (113 of 121) were associated with only one environmental variable.

Characterizing significant SNP loci based on their relationship with known protein‐coding genes showed that 28 environment‐associated loci were located within the identified sequence of a gene, corresponding to 26 unique proteins. Functional annotation showed that the identified genes are involved in morphology and growth, neurodevelopment, and metabolism (Table 3).

**TABLE 3:** Genes found to be under selection using environmental association analysis with corresponding functional annotation

Environmental predictor	Gene name	Annotation	Notable references in other fish
Predator index	LOC103462904	Crucial for migration, repulsion and adhesion during neuronal, vascular, and epithelial development. Also involved in the immune response	
	LOC108166987	n/a	
	gna12	Involved in G protein‐coupled receptor binding and dopamine receptor binding. Coordinates cell migration during in gastrulation, growth factors signaling to cell surface receptors, and actin turnover in various processes in the cells including cytoskeleton formation	Sarwal et al. (1996)
	sorbs2	Functions in signaling complexes as a link between ABL kinases and the actin cytoskeleton. Involved in cell growth during cardiac muscle cell development	
	LOC103462142	n/a	
Landscape PC1 (percent forest, urbanization)	LOC103475011	Thyroid hormone transmembrane transporter activity	
	LOC103474479	Functions in calcium‐binding	
	cacna1a	Mediates entry of calcium ions into excitable cells. Also involved in a variety of calcium‐dependent processes, including muscle contraction, hormone or neurotransmitter release, gene expression, cell motility, cell division, and cell death	Schunter et al. (2018)
Landscape PC1 (watershed slope, area and percent agriculture	LOC103474897	n/a	
	LOC103474183	Microtubule‐based processes	
	zfyve27	Neuron projection development. May be a sex‐determining gene in fish	
	xylt1	Embryonic cranial skeleton morphogenesis	Eames et al. (2011)
	LOC103464456	Protein transport	
	LOC103464222	Ion transport	
	cacna1a	Mediates entry of calcium ions into excitable cells. Also involved in a variety of calcium‐dependent processes, including muscle contraction, hormone or neurotransmitter release, gene expression, cell motility, cell division, and cell death	Schunter et al. (2018)
	adamts1	n/a	Liu et al. (2018)
	LOC103462875	Phospholipid metabolism	
	LOC103462875	Phospholipid metabolism	
	pla2g6	Lipid catabolism	Sánchez et al. (2018)
	LOC103463029	n/a	
	zfyve27	Neuron projection development	
	LOC103478748	Functions in the Wnt signaling pathway during embryonic development	
	gde1	Lipid metabolism	Garcia‐Reyero et al. (2011)
	ubl5	mRNA processing	
	LOC103470565	ADP‐ribosylation	
Water quality PC1 (nitrogen)	radil	Underlies multicellular organism development and forms neural crest precursors	
Water quality PC2 (phosphorus)	caskb	Regulates neurotransmitter release, axon branching and dendritic outgrowth, and stabilizes morphology. Interacts with neurexins in synapse development and activity. Exhibits protein kinase activity and plays a role in calcium metabolism that is altered by uranium exposure	Lerebours et al. (2010), Rissone et al. (2007)
	nacc1	Acts as a transcriptional corepressor in neuronal cells. Required for recruiting the proteasome and responsive to pollution	

## 4 DISCUSSION

### 4.1 Population structure and invasion history

Our findings indicate that there is significant genomic variation and structure among guppy populations across the Hawaiian archipelago. The development of population structure is consistent with evidence of relatively little gene flow among populations. We found that there is a relatively higher proportion of molecular variance between populations within each island than among islands, which indicates that genetic drift overwhelms any ongoing gene flow among watersheds. Notably, divergence modeling of Maui and O‘ahu was best explained by a model without gene flow. In contrast, *D*‐statistics clearly indicate an excess of shared alleles between populations on the island of O‘ahu with populations from Maui, Kaua‘i, and Hawai‘i suggestive of gene flow (Table 1). Among trios including populations from three different islands, *D*‐statistics were only significant for trios including populations from both O‘ahu and Maui, so the relationships between populations on Maui, Kaua‘i, and Hawai‘i can be adequately represented by a traditional bifurcating tree. If gene flow from O‘ahu to Hawai‘i and Kaua‘i occurred after their split from Maui, we would expect our observed pattern of significant *D*‐statistic values for O‘ahu‐Maui‐Kaua‘i and O‘ahu‐Maui‐Hawai‘i trios and the demographic modeling result of no migration between O‘ahu and Maui. While *D*‐statistics cannot be used to resolve the direction of gene flow, movement of guppies from Maui and O‘ahu to Kaua‘i and the island of Hawai‘i is more probable than movement in the opposite direction. This interpretation is supported by the grouping of Maui populations with Trinidadian native range populations in the admixture graph analyses and the historical accounts indicating O‘ahu as the initial point of introduction to the Hawaiian archipelago (Brock, 1960). Furthermore, it is consistent with how the admixture graph analyses (Figure 3) represent relationships among watersheds for each island. The limited gene flow within each island, as inferred from *D*‐statistics, suggests that human transport of individuals between watersheds is not a frequent occurrence. While guppies can tolerate high‐salinity conditions in a laboratory setting (Chervinski, 1984), the combination of traversing both a marine environment and large in‐stream barriers (e.g., waterfalls) likely restricts guppy movement.

Patterns of genomic variation indicate a more complicated invasion history than suggested by historical records (Brock, 1960). Demographic modeling results indicate that the invasion of guppies to the Hawaiian archipelago started with an introduction from a single source to Maui and/or O‘ahu. Individuals from these islands were then translocated to the islands of Kaua‘i and Hawai‘i. Populations likely experienced drift throughout this process, allowing genetic divergence to accumulate. Estimates of population structure support this inference; guppies on the islands of Kaua‘i and Hawai‘i, in contrast to O‘ahu and Maui, show evidence of a more admixed population history (Figure 2, Figure S1). The inference of a single introduction source should be robust to unsampled populations. As there is substantial regional population structure in native range guppy populations (Alexander et al., 2006), one or more of our analyses would likely have indicated that Hawaiian guppies were derived from multiple native range populations, even if those populations were not included for reference. Any ancestral variation would inflate both the *D*‐statistics (Peter, 2016) and alter the admixture graph for the affected populations. Notably, a similar pattern of differentiation was found in non‐native nematode parasites that infect native goby species but originally reached the archipelago through co‐introduction with guppies and their relatives from the Americas; patterns of genetic variation and demographic reconstructions indicate that the parasites were originally introduced to O‘ahu and spread to Maui before spreading to other islands (Gagne et al., 2018). Though we have inferred that only one source population was introduced to the archipelago, we cannot determine its precise source; guppies have been transported worldwide from South America and the Lesser Antilles, and Hawaiian guppies could be descended from one of many native range, captive, or established invasive populations. Because our results do not support introductions from multiple source populations to the Hawaiian archipelago, the observed genetic variation must represent a combination of genetic diversity within the initial founding populations and population divergence since introduction.

### 4.2 Adaptation to novel environments

Guppies are renowned for their ability to rapidly adapt to novel predation regimes (Gordon et al., 2009; Reznick et al., 1990, 1997), which raises the possibility that other ecological gradients might elicit similar adaptive responses. Consistent with this idea, we identified SNP loci associated with all five of the tested environmental variables. The loci associated with three of our environmental variables exhibited an enriched number of extreme allele frequency shifts across the corresponding environmental gradients. Interestingly, weaker allele frequency shifts were observed in association with the principle components reflecting urban development and nitrogen pollution (landscape PC1, water quality PC1; Lisi et al., 2018), which correspond to “urban stream syndrome” conditions. This suggests that these variables may not impose as much selection on guppy populations as other environmental factors, or at least that responses of guppies to these conditions involve subtle allele frequency change at the loci we surveyed. Other studies have shown that guppies can persist in degraded (Lisi et al., 2018) and polluted (Araújo et al., 2009) environments, in part by modifying their behavior in response to low oxygen concentrations and water quality (Kramer & Mehegan, 1981).

The presence of genetic environmental associations in Hawaiian guppy populations should be interpreted in light of demographic patterns, as substantial genetic drift could inflate false‐positive rates in these statistical tests (Schoville et al., 2012). The stairway plot analyses estimate the effective population sizes of both native and non‐native range guppy populations in hundreds of individuals, despite founder effects. When paired with large census population sizes (densities as high as 11.0 guppies/m^2^; Lisi et al., 2018), it is not unreasonable to think that natural selection could overcome the effects of genetic drift at some loci in Hawaiian guppy populations.

It is worth noting that less than 25% of our environmentally associated loci were linked to a specific protein. Although other significant loci were neither within nor very close to a protein‐coding gene, they could still be physically linked with or regulate transcription of a nearby gene associated with local adaptation. Based on previous work, the genes in which environmentally associated loci were found show some noteworthy patterns. One gene, associated with landscape PC2, was determined to be involved in a plastic response to changes in predation levels in Trinidadian guppy populations (Ghalambor et al., 2015). Furthermore, 13 of the 26 genes associated with at least one environmental metric were found to be differentially expressed between male and female guppies in the brain, tail, or gonadal tissue (Sharma et al., 2014). Sharma et al. (2014) found increased rates of coding sequence evolution in genes with sex‐biased expression, which could explain why they constitute half of our environmentally correlated genes. Elevated rates of molecular evolution are hypothesized to be driven by sexual selection, sex‐biased selection, or relaxed purifying selection (Mank & Ellegren, 2009; Parsch & Ellegren, 2013). It is well known that guppies are under strong sexual selection pressures (Houde, 1987; Houde & Endler, 1990; Kodric‐Brown & Nicoletto, 2001) and that natural selection can act on sexually selected characteristics (Endler, 1991). Sex‐specific selective pressures are associated with predation and intraspecific communication (e.g., light environment) in streams (Archard et al., 2009; Endler, 1991), but we cannot determine whether our suite of environmental variables directly or indirectly reflects differences in associated conditions across streams. Finally, functional annotation revealed that many of the putatively selected genes play a role in morphology and growth, neurodevelopment, and metabolism (Table 3). Together, the observed patterns highlight the possibility that stream‐level variation in predator community and environmental conditions drive local adaptation in Hawaiian guppies. Further work to test this hypothesis should include an examination of morphological differences among populations and reciprocal transplant experiments to verify that the loci identified here convey a fitness advantage respective to local environment. To complement this, a higher density of genomic data could facilitate a better understanding of the mechanisms by which our identified loci increase fitness.

We found little evidence for genetic signatures of parallel adaptation to predation regime in Hawaiian and Trinidadian guppies. Most of the alleles associated with adaptation to higher levels of predation in Hawaiian populations are not represented in both of our high‐predation Trinidadian populations. This contrasts with patterns of parallel genetic evolution in sticklebacks that have evolved from common source populations (Cresko et al., 2004; Hohenlohe et al., 2010) and suggests that guppies in the Hawaiian archipelago may not be derived from Trinidadian populations or that guppies have multiple means of adapting to high predation pressure. Comparing life‐history traits (e.g., size and age at maturity, average offspring size) could confirm whether Hawaiian and Trinidadian guppies have used different loci to attain similar phenotypes when adapting to differences in predation regime.

Surprising evolutionary parallels can be drawn between guppies and native Hawaiian fishes. All of the native fishes in Hawaiian streams are amphidromous; thus, high gene flow resulting from marine larval dispersal should preclude local adaptation. Yet, it has been shown that one native species, *Sicyopterus stimpsoni*, adaptively evolves in response to heterogeneous selective pressures resulting from predation and landscape characteristics (Moody et al., 2015). Phenotypic differences appear to be maintained by high levels of predation, with differences in predation pressure across watersheds being associated with watershed slope (Moody et al., 2019). There is evidence for a similar pattern in Hawaiian guppies, as watershed slope is a significant constituent of our landscape PC2 metric, which was associated with the highest number of loci out of all tested variables (Lisi et al., 2018). It is important to recognize, however, that responsiveness to differences in watershed slope could instead reflect sensitivity to variation in other environmental factors including surface water velocity, which has been associated with morphological divergence in guppies (Hendry et al., 2006).

The limited dispersal capability of guppies, particularly compared to the movement potential of amphidromous native stream fishes of the Hawaiian archipelago, has likely facilitated rapid adaptation. Adaptive evolution in *S*. *stimpsoni* appears to be the result of strong selection (Moody, Kawano, et al., 2017) constrained by archipelago‐wide gene flow (Moody et al., 2019). In contrast, any potential local adaptation in the Hawaiian guppy populations may proceed rapidly in response to weaker selective pressures, in part due to life‐history and geographic constraints to movement between watersheds. These conditions are more akin to those that have given rise to adaptive radiations of terrestrial Hawaiian biota, such as *Drosophila*, silverswords, honeycreepers, and spiders (Gillespie et al., 1994; Lerner et al., 2011; Montgomery, 1975; Witter & Carr, 1988) than native Hawaiian stream fishes (Moody et al., 2015, 2019; Moody, Kawano, et al., 2017).

Our findings raise the possibility that local adaptation is contributing to the success of guppies in Hawaiian streams. As anthropogenic change continues to alter and increase variability among Hawaiian watersheds, the benefits imparted by readily adapting to local conditions may outweigh the (meta‐)population stability imparted by a migratory life history. Nearly, all native fishes fare poorly in degraded streams (Blum et al., 2014; Brasher, 2003; Lisi et al., 2018; Moody, Gagne, et al., 2017), though there is some evidence that species with dietary and migration flexibility are more capable of coping with degradation (Hogan et al., 2014; Lisi et al., 2018). Further studies using experimental selection trials, reciprocal transplants, and common garden experiments will advance our understanding of local adaptation in native and non‐native Hawaiian stream fishes. Additionally, these and other studies could assess the intriguing possibility that ecological and life‐history flexibility confers greater capacity for local adaptation in native Hawaiian fishes.

### 4.3 Implications for conservation management

Establishment and spread of non‐native species can depend on a range of factors, such as the number of individuals introduced, tolerance (i.e., pre‐adaptation) for a new environment and biotic interactions with native species, and the propensity for rapid adaptation (Bruno et al., 2005; Jones & Gomulkiewicz, 2012). Our results suggest that the invasion of guppies across the Hawaiian archipelago has been facilitated by life‐history characteristics and relatively large founding populations. For example, viviparity and a short generation time have likely contributed to the postintroduction spread of guppies. A short generation time might have resulted in rapid population expansion after a relatively short lag period of population growth, even if a stream was seeded with a small number of individuals. Adaptive evolution might also act jointly with life‐history attributes to increase the likelihood of establishment and persistence in newly founded populations.

We observed evidence of adaptive variation in genes linked to growth, development, and metabolism. The single introduction to the archipelago and short time span implies that any adaptive evolution in Hawaiian populations likely utilized the standing diversity in large founder populations. Our results also suggest that different ecological factors can drive local adaptation in freshwater biological invasions, which affords a stronger basis for developing management strategies for controlling populations and limiting spread. Additionally, our findings point to the potential value of removal as a method for controlling guppies in Hawaiian streams. Due to low movement among watersheds, we can infer that natural recolonization by guppies is unlikely if locally adapted populations can be successfully removed from streams that are a high conservation priority (Moody et al., 2021). Guppy introductions have been linked to the spread of non‐native nematode parasites (Gagne et al., 2015), declines in endemic damselflies (Englund & Museum, 1999), shifts in the behavior of some native invertebrates (e.g., *Halocaridina rubra*; Havird et al., 2014), and declines in native fishes (Blum et al., 2014; Hain et al., 2019; Holitzki et al., 2013). However, some stakeholders may value guppies for their perceived ecosystem benefits such as mosquito control, even though guppies are not an effective mechanism for controlling mosquitoes and they are demonstrably damaging to the ecology of local ecosystems (El‐Sabaawi et al., 2016; Englund & Museum, 1999; Holitzki et al., 2013). Thus, extirpating guppies from a watershed requires outreach and stakeholder input (Blum et al., 2020). The need for community engagement would be important to consider for managing any invasive species that stakeholders perceive a benefit from, but especially for species such as guppies that can establish rapidly even with minimal propagule inputs.

## CONFLICT OF INTEREST

The authors declare no conflicts of interest.

## Supporting information

Appendix S1

Appendix S2

## Data Availability

Short read data have been deposited in NCBI GenBank in the short read archive (Bioproject: PRJNA592560, accessions: SRR10572011–SRR10572014). Mitochondrial control region data have been deposited in NCBI as accessions: MN922313–MN922320.
