# Supplementary material for: Invasion and rapid adaptation of guppies (Poecilia reticulata) across the Hawaiian Archipelago
Source: Evol Appl. 2021 May 4;14(7):1747–61. doi: 10.1111/eva.13236 (PMC8288002; doi:10.1111/eva.13236)
Supplement: Supplementary file 1 — Appendix S1 [file EVA-14-1747-s001.docx]

**Supplementary File 1**

**Table S1:** Sampling information and statistics of genetic diversity for each sampled guppy population, as measured by expected multilocus heterozygosity (H_e_) and nucleotide diversity (π).

| **Population** | **Region** | **Latitude** | **Longitude** | **Individuals** | **H_e_** | **π** |
| --- | --- | --- | --- | --- | --- | --- |
| 22004 | HI - Kaua‘i | 22.10502778 | -159.3281944 | 29 | 0.0026 | 0.0028 |
| 22013 | HI - Kaua‘i | 21.97580556 | -159.3740278 | 14 | 0.0026 | 0.0029 |
| 23004 | HI - Kaua‘i | 21.9209444 | -159.507444 | 9 | 0.0035 | 0.004 |
| 31018 | HI - O‘ahu | 21.5350833 | -157.888722 | 13 | 0.0026 | 0.0028 |
| 32002 | HI - O‘ahu | 21.49222 | -157.85088 | 13 | 0.0023 | 0.0026 |
| 32004 | HI - O‘ahu | 21.48009 | -157.86078 | 13 | 0.0055 | 0.0059 |
| 32007 | HI - O‘ahu | 21.45277778 | -157.8351944 | 11 | 0.0018 | 0.0022 |
| 32008 | HI - O‘ahu | 21.41009 | -157.82266 | 12 | 0.0018 | 0.002 |
| 33007 | HI - O‘ahu | 21.29108333 | -157.8146944 | 10 | 0.0037 | 0.0042 |
| 33011 | HI - O‘ahu | 21.36485 | -157.84166 | 15 | 0.0018 | 0.002 |
| 34002 | HI - O‘ahu | 21.38349 | -157.89941 | 13 | 0.0019 | 0.0022 |
| 61001 | HI - Maui | 20.81104 | -156.58661 | 14 | 0.0019 | 0.0021 |
| 61011 | HI – Maui | 20.997598 | -156.603734 | 15 | 0.0018 | 0.002 |
| 62007 | HI – Maui | 20.942723 | -156.530521 | 15 | 0.0018 | 0.002 |
| 62009 | HI – Maui | 20.88075 | -156.54439 | 14 | 0.0021 | 0.0024 |
| 82049 | HI - Hawai‘i | 19.79586 | -155.09395 | 14 | 0.002 | 0.0023 |
| 82061 | HI - Hawai‘i | 19.71505556 | -155.0793889 | 3 | 0.0028 | 0.0033 |
| 85003 | HI – Hawai‘i | 20.01386 | -155.825 | 15 | 0.0023 | 0.0026 |
| Yarra | Trinidad | 10.740861 | -61.321111 | 12 | 0.001 | 0.0011 |
| Quare | Trinidad | 10.662944 | -61.192778 | 12 | 0.0015 | 0.0018 |
| Aripo | Trinidad | 10.6505 | -61.223194 | 12 | 0.0017 | 0.0021 |
| Rio las Marias | Venezuela | 9.2 | -69.71 | 4 | 0.0007 | 0.0012 |

**Table S2:** Pairwise F_ST_ values for each sampled guppy population. The column and row labels are population names.

|  | HI - O‘ahu | HI - Kaua‘i | HI - O‘ahu | HI - Kaua‘i | HI - Kaua‘i | HI – Maui | HI – Maui | HI - Hawai‘i | HI - O‘ahu | HI - O‘ahu | HI - O‘ahu | HI – Maui | HI – Maui | HI - Hawai‘i | HI - O‘ahu | HI - Hawai‘i | HI - O‘ahu | HI - O‘ahu | Trinidad | Trinidad | Trinidad | Venezuela |
| --- | --- | --- | --- | --- | --- | --- | --- | --- | --- | --- | --- | --- | --- | --- | --- | --- | --- | --- | --- | --- | --- | --- |
|  | **32004** | **23004** | **31018** | **22013** | **22004** | **62009** | **62007** | **85003** | **33007** | **34002** | **32008** | **61011** | **61001** | **82049** | **33011** | **82061** | **32002** | **32007** | **Yarra** | **Quare** | **Aripo** | **Rio las Marias** |
| **32004** | 0 |  |  |  |  |  |  |  |  |  |  |  |  |  |  |  |  |  |  |  |  |  |
| **23004** | 0.1388 | 0 |  |  |  |  |  |  |  |  |  |  |  |  |  |  |  |  |  |  |  |  |
| **31018** | 0.1607 | 0.2148 | 0 |  |  |  |  |  |  |  |  |  |  |  |  |  |  |  |  |  |  |  |
| **22013** | 0.0799 | 0.0793 | 0.1663 | 0 |  |  |  |  |  |  |  |  |  |  |  |  |  |  |  |  |  |  |
| **22004** | 0.1150 | 0.0842 | 0.1920 | 0.0534 | 0 |  |  |  |  |  |  |  |  |  |  |  |  |  |  |  |  |  |
| **62009** | 0.2048 | 0.1409 | 0.2644 | 0.1490 | 0.1541 | 0 |  |  |  |  |  |  |  |  |  |  |  |  |  |  |  |  |
| **62007** | 0.2087 | 0.1477 | 0.2719 | 0.1508 | 0.1534 | 0.1669 | 0 |  |  |  |  |  |  |  |  |  |  |  |  |  |  |  |
| **85003** | 0.1627 | 0.1184 | 0.2294 | 0.1205 | 0.1335 | 0.1823 | 0.1757 | 0 |  |  |  |  |  |  |  |  |  |  |  |  |  |  |
| **33007** | 0.0553 | 0.0828 | 0.1393 | 0.0443 | 0.0732 | 0.1582 | 0.1619 | 0.1155 | 0 |  |  |  |  |  |  |  |  |  |  |  |  |  |
| **34002** | 0.0937 | 0.1491 | 0.1818 | 0.1020 | 0.1189 | 0.2157 | 0.2168 | 0.1698 | 0.0716 | 0 |  |  |  |  |  |  |  |  |  |  |  |  |
| **32008** | 0.1016 | 0.1555 | 0.1701 | 0.1010 | 0.1241 | 0.2160 | 0.2091 | 0.1728 | 0.0777 | 0.1066 | 0 |  |  |  |  |  |  |  |  |  |  |  |
| **61011** | 0.2232 | 0.1728 | 0.2928 | 0.1714 | 0.1772 | 0.2016 | 0.1902 | 0.2020 | 0.1792 | 0.2328 | 0.2311 | 0 |  |  |  |  |  |  |  |  |  |  |
| **61001** | 0.1880 | 0.1133 | 0.2563 | 0.1320 | 0.1402 | 0.0915 | 0.1614 | 0.1676 | 0.1485 | 0.1947 | 0.2052 | 0.1942 | 0 |  |  |  |  |  |  |  |  |  |
| **82049** | 0.1398 | 0.1458 | 0.2052 | 0.1214 | 0.1434 | 0.1962 | 0.1985 | 0.1004 | 0.0935 | 0.1574 | 0.1395 | 0.2140 | 0.1913 | 0 |  |  |  |  |  |  |  |  |
| **33011** | 0.0830 | 0.1618 | 0.1869 | 0.1043 | 0.1295 | 0.2242 | 0.2261 | 0.1835 | 0.0726 | 0.1032 | 0.1057 | 0.2367 | 0.2138 | 0.1570 | 0 |  |  |  |  |  |  |  |
| **82061** | 0.1655 | 0.1738 | 0.2334 | 0.1466 | 0.1574 | 0.2320 | 0.2477 | 0.1260 | 0.1166 | 0.1911 | 0.1742 | 0.2512 | 0.2187 | 0.0825 | 0.1776 | 0 |  |  |  |  |  |  |
| **32002** | 0.0800 | 0.1644 | 0.1681 | 0.1056 | 0.1339 | 0.2235 | 0.2237 | 0.1831 | 0.0773 | 0.1163 | 0.1247 | 0.2430 | 0.2142 | 0.1613 | 0.1247 | 0.1874 | 0 |  |  |  |  |  |
| **32007** | 0.0496 | 0.1215 | 0.1695 | 0.0705 | 0.1044 | 0.1854 | 0.1901 | 0.1473 | 0.0537 | 0.1060 | 0.1023 | 0.2127 | 0.1635 | 0.1310 | 0.0860 | 0.1649 | 0.1139 | 0 |  |  |  |  |
| **Yarra** | 0.5189 | 0.5307 | 0.5558 | 0.4921 | 0.4575 | 0.5371 | 0.5542 | 0.5088 | 0.5091 | 0.5376 | 0.5511 | 0.5583 | 0.5288 | 0.5108 | 0.5303 | 0.6233 | 0.5323 | 0.5300 | 0 |  |  |  |
| **Quare** | 0.3377 | 0.3258 | 0.3953 | 0.3052 | 0.2950 | 0.3574 | 0.3708 | 0.3319 | 0.3109 | 0.3524 | 0.3594 | 0.3820 | 0.3430 | 0.3305 | 0.3572 | 0.4134 | 0.3542 | 0.3306 | 0.5664 | 0 |  |  |
| **Aripo** | 0.2333 | 0.2094 | 0.3037 | 0.2005 | 0.2035 | 0.2590 | 0.2649 | 0.2358 | 0.1993 | 0.2508 | 0.2538 | 0.2827 | 0.2401 | 0.2342 | 0.2529 | 0.2891 | 0.2559 | 0.2247 | 0.5025 | 0.2914 | 0 |  |
| **Rio las Marias** | 0.3023 | 0.2892 | 0.3618 | 0.2674 | 0.2633 | 0.3287 | 0.3504 | 0.2958 | 0.2686 | 0.3238 | 0.3307 | 0.3593 | 0.3224 | 0.3006 | 0.3241 | 0.3763 | 0.3245 | 0.3020 | 0.6168 | 0.4221 | 0.2994 | 0 |
|  |  |  |  |  |  |  |  |  |  |  |  |  |  |  |  |  |  |  |  |  |  |  |


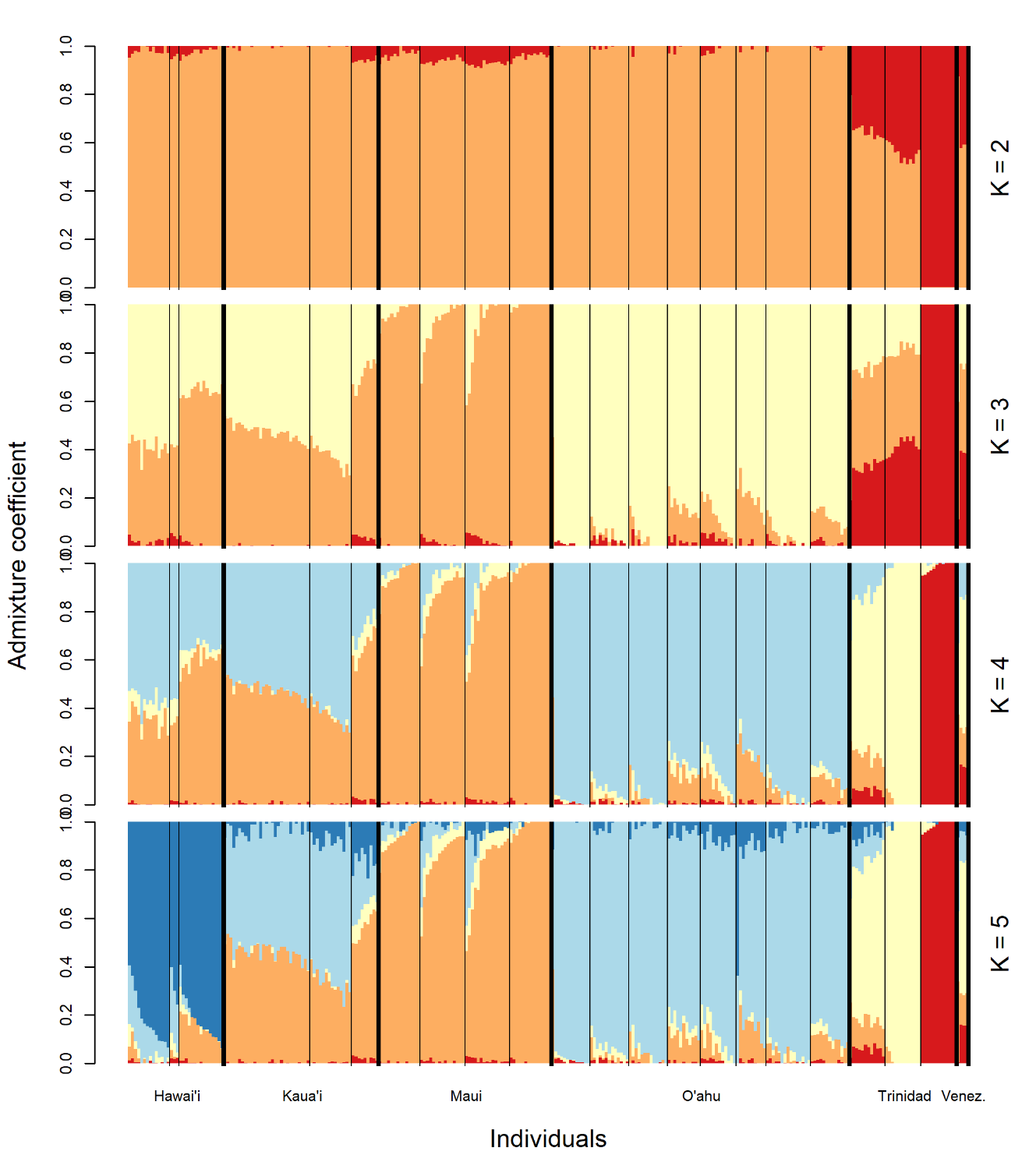


**Figure S1:** Plots of sNMF results for values of K=2 though K=5. Each thin black line separates watersheds within an island or region, and each island or region is separated by a thick black line.


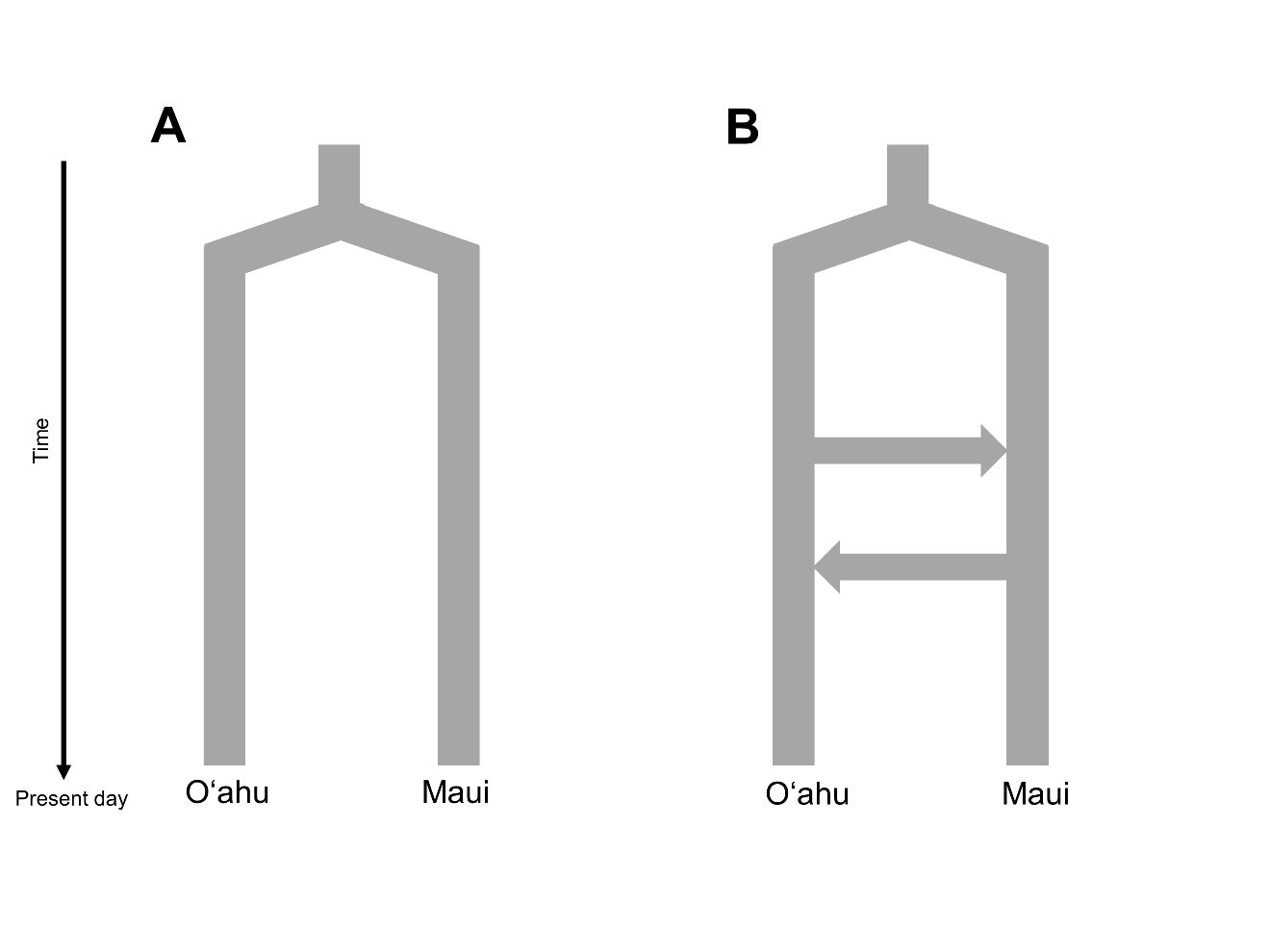


**Figure S2:** Conceptual diagrams of both demographic models used in the analysis of population invasion history. Model A shows the divergence of O‘ahu and Maui without gene flow, whereas Model B shows the same but with continuous gene flow (allowed to be asymmetrical) between the two after divergence. Model A was nested within Model B.


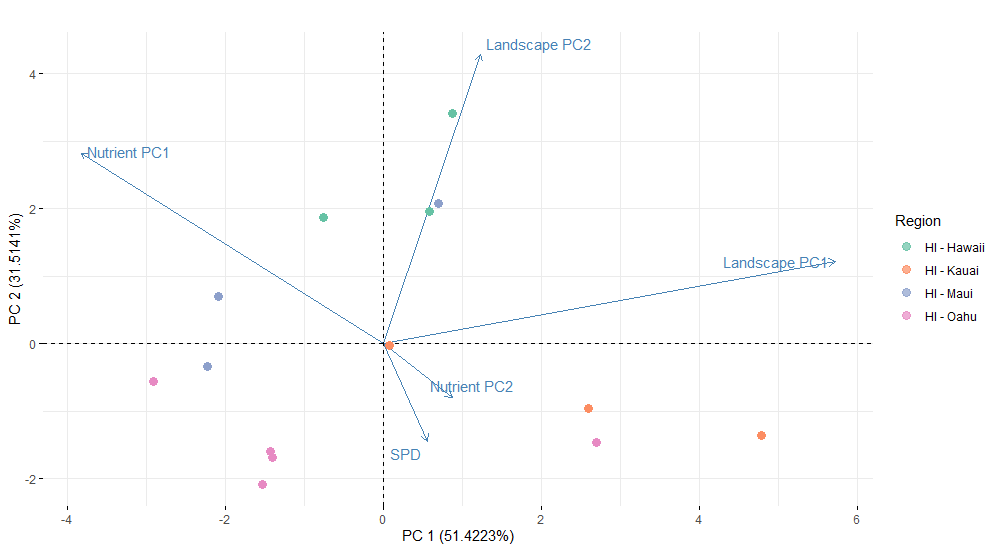


**Figure S3:** Plot of PCA results for watershed environmental data from the sampled Hawaiian populations that were not missing any environmental data. Each point represents a sampled watershed, and points are colored by the island of origin. Lines indicate the loading of each environmental variable on PC scores. Axes labels indicate the percent of total variance explained by each principal component.


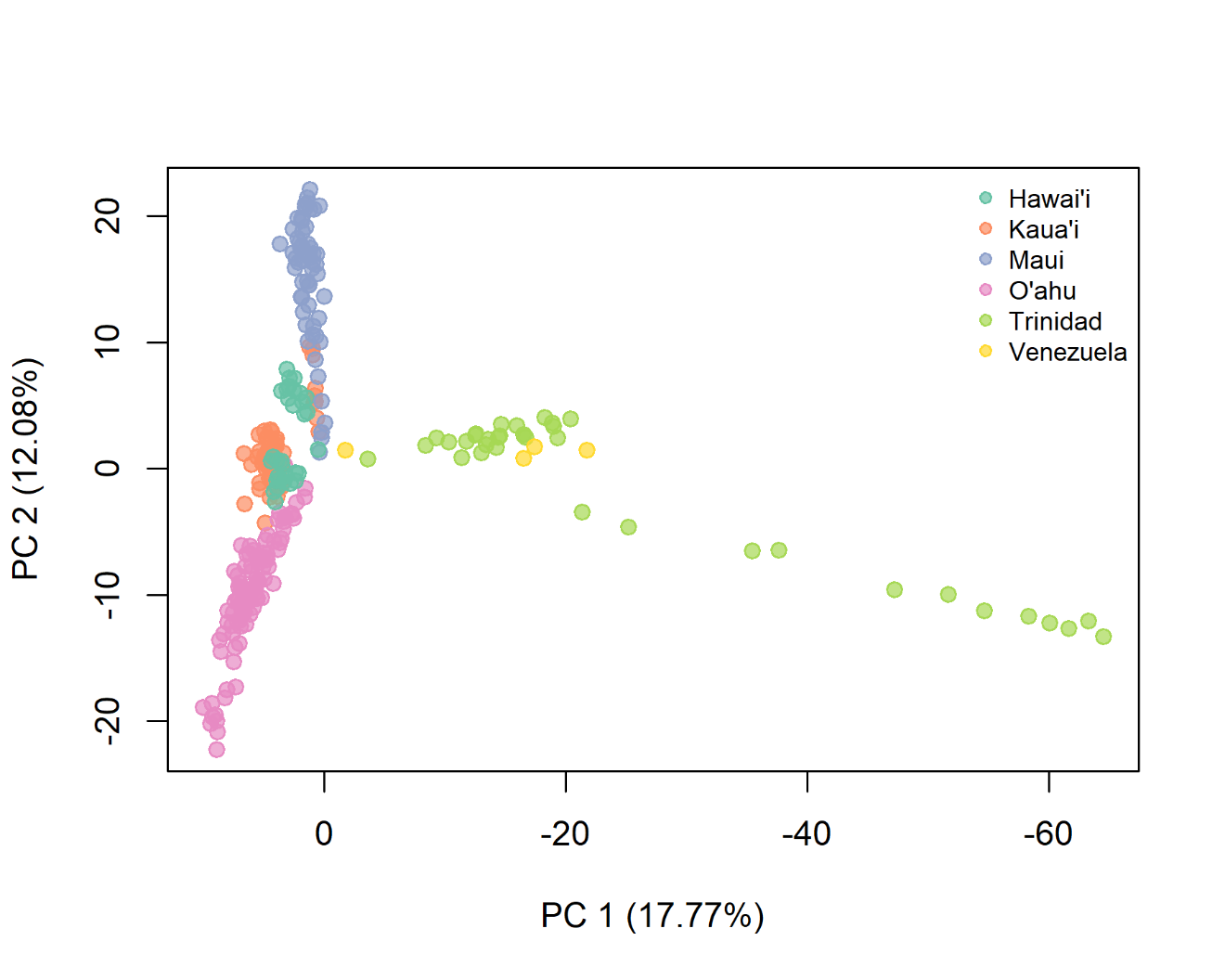


**Figure S4:** Plot of PCA results for guppy genotype data before genotype imputation. Points are colored by their region (island or country) of origin. Results show a similar pattern of population structuring to that observed in Figure 1A, but with “smearing” of populations (especially for Trinidadian populations) often seen in PCA done with higher amounts of missing data. Axes labels indicate the percent of total variance explained by each principal component.

**
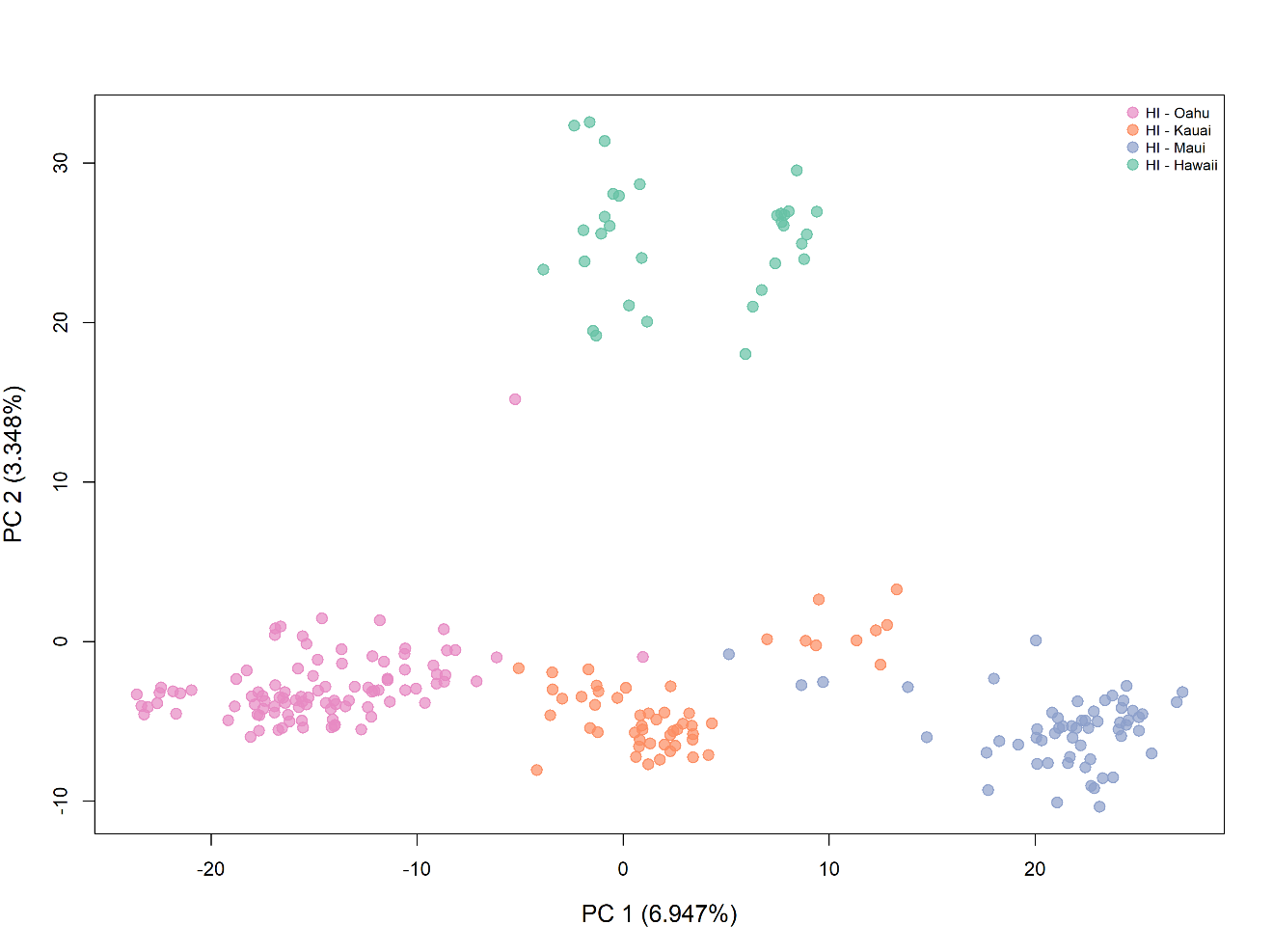
**

**Figure S5:** Plot of PCA results for guppy genotype data from Hawaiian individuals. Points are colored by their region (island or country) of origin. Axes labels indicate the percent of total variance explained by each principal component.


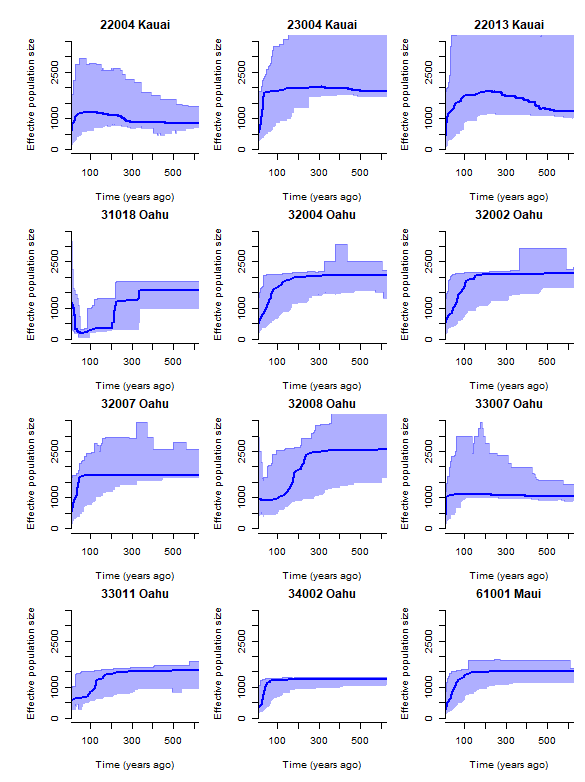


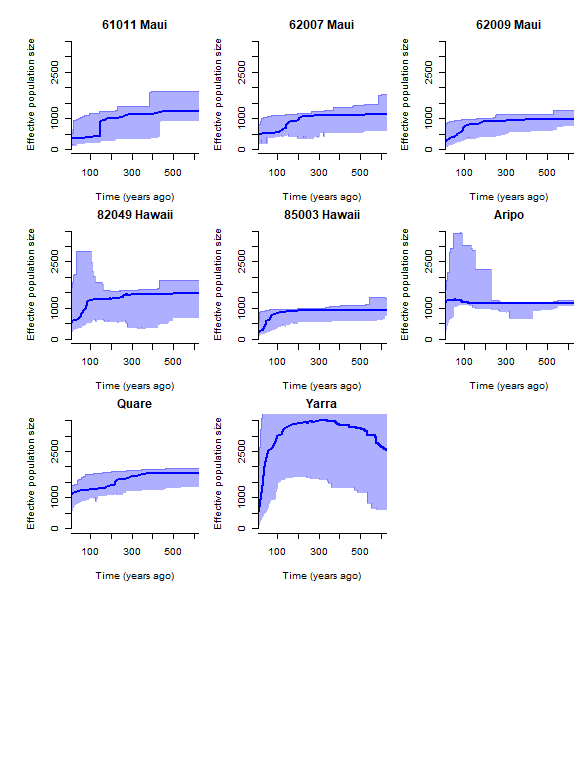


**Figure S6:** Stairway plot results, including 95% confidence intervals for the sampled Hawaiian and native range guppy populations. Each plot shows change in inferred effective populations size (N_e_) over time. Many populations show a decrease in the recent past.

**
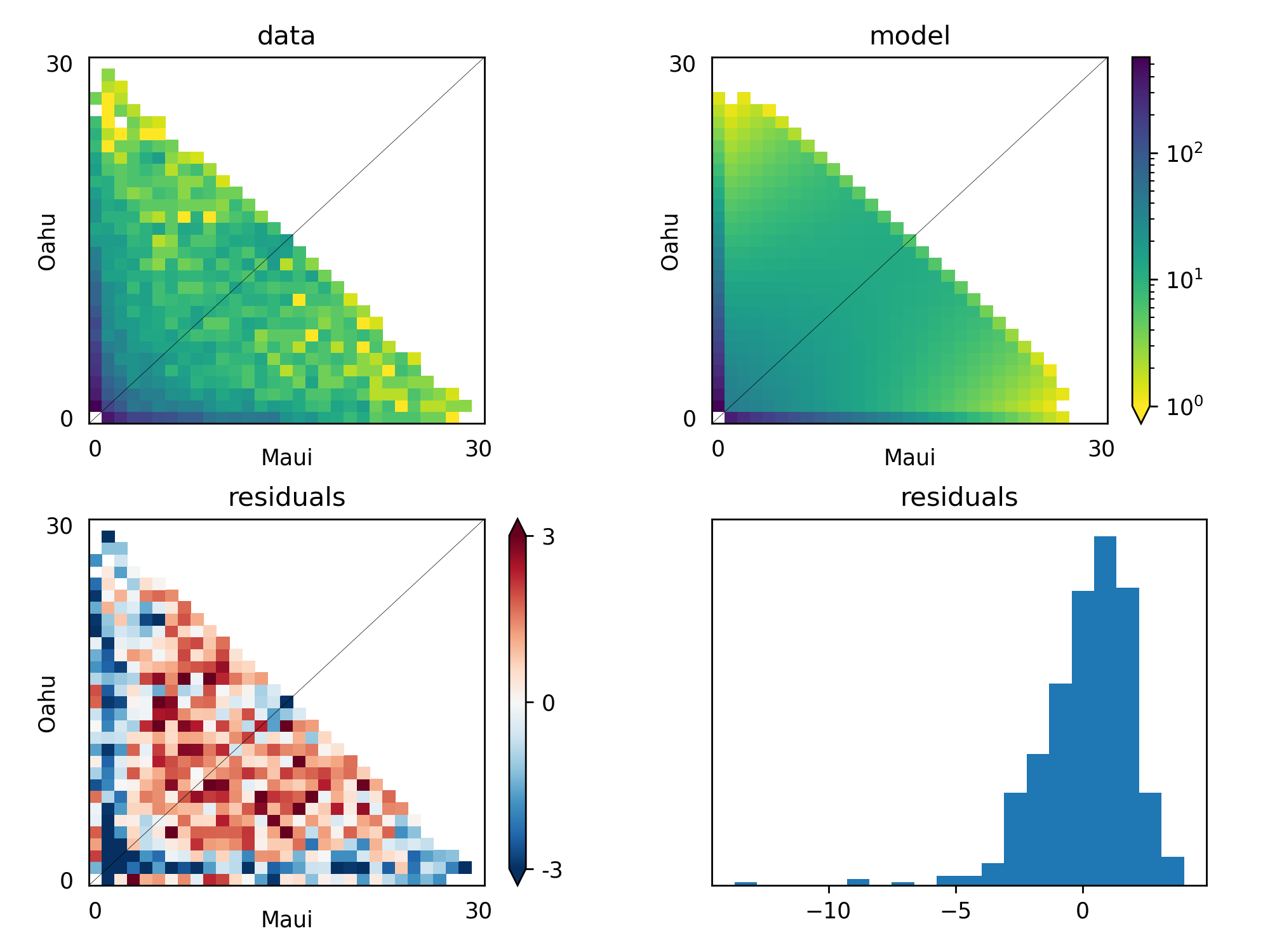
**

**Figure S7:** Plot of dadi results for the two-population divergence model without migration between O‘ahu (represented by population 33011) and Maui (represented by population 62007). The windows of the plot show the 2-dimensional frequency spectra for the model and data, as well as the residuals between the model and the data.
